# A Modified Protocol for the Isolation, Culture, and Characterization of Human Smooth Muscle Cells from the Umbilical Cord

**DOI:** 10.3390/mps6030054

**Published:** 2023-05-26

**Authors:** Asmaa Q. Ibrahim, Mohammed S. Abdullah, Mamoun Ahram, Shtaywy Abdalla

**Affiliations:** 1 Department of Biological Sciences, School of Science, The University of Jordan, Amman 11942, Jordan; 2 Department of Physiology and Biochemistry, School of Medicine, The University of Jordan, Amman 11942, Jordan

**Keywords:** isolation protocol, vascular smooth muscle cells, VSMC characterization, umbilical cord

## Abstract

Background: Vascular smooth muscle cells (VSMCs) and vascular endothelial cells are key participants in the pathogenesis of atherosclerosis. Human umbilical vein endothelial cells (HUVECs) and VSMCs are useful models to design therapeutic strategies for many cardiovascular diseases (CVDs). However, procuring a VSMC cell line by researchers, to model atherosclerosis, for example, is impeded by time and cost limitations, as well as by many other logistic problems in many countries. Results: This article describes a protocol for the quick and cheap isolation of VSMCs from human umbilical cords using a mechanical and enzymatic method. This VSMC protocol yields a confluent primary culture that could be obtained within 10 days and sub-cultured for 8–10 passages. The isolated cells are characterized by their morphology and the expression of mRNA of marker proteins analyzed by reverse transcription polymerase chain reaction (RT-qPCR). Conclusion: The protocol described herein for the isolation of VSMCs from human umbilical cords is easy and is time- and cost-efficient. Isolated cells are useful models for understanding the mechanisms underlying many pathophysiological conditions.

## 1. Introduction

Atherosclerosis is the pathological basis of peripheral vascular, coronary, and cerebrovascular diseases and is a major cause of mortality and morbidity [1]. Endothelial injury, lipid deposition, inflammation, modulation of vascular smooth muscle cell phenotypes, migration, and hypertrophy are all key events in vascular diseases [2]. Studies employing in vitro cultures of human umbilical vein endothelial cells (HUVECs) and vascular smooth muscle cells (VSMCs) have enriched the understanding of this disease and others [3,4].

To perform in vitro research on VSMCs, researchers usually resort to procuring cell lines from an accredited commercial biological supply house, a research center, or other researchers. A disadvantage of purchasing cell lines from reputable suppliers is the time required for shipping, customs clearance, and cost, especially with institutions that suffer a shortage of funds. Shipping restrictions and bureaucracies may significantly impede the purchase of cell lines. Moreover, cell line misidentification and contamination with microorganisms, such as mycoplasma, together with improper shipping and customs handling would result in cell instability or even death. For all these reasons, cell isolation may be a better choice. Therefore, the existence of an optimized protocol for isolating VSMCs in a reasonable time and at a reasonable cost would offer a continuous supply of cells.

The umbilical cord is a readily available source of HUVECs and VSMCs. A particular form of unnerved adventitia, called Wharton’s jelly, is formed in the umbilical cord by mucous connective tissue and fibroblasts that surround the blood vessels [5]. It also has two arteries and one vein. The walls of both arteries and veins have three layers: tunica intima, tunica media, and tunica adventitia. In arteries, the tunica intima is separated from the media by an internal elastic lamina that forms the most external component of the intima [6]. The tunica media consists chiefly of concentric layers of helically arranged smooth muscle cells. The media in the arteries is also separated from the tunica adventitia by a thin external elastic lamina. The tunica adventitia consists principally of fibroblasts with type I collagen and elastic fibers [7].

Two methods were generally used for isolating VSMCs, either by allowing cells to migrate from an explanted medial tissue or by enzymatically dispersing the cells from the tunica media [4,8]. One limitation of the explant method is that it takes 2–3 weeks of culturing until cells start to grow out of the explant, and it is difficult to handle and maintain the explant attached to the ground of the cell culture dish throughout this period [8]. On the other hand, an important limitation of the enzymatic dispersion method is the low yield of cells compared to the explant method.

Another important point to consider is the phenotypic change in or plasticity of the VSMCs [4]. Both in vivo and in vitro VSMCs can develop either contractile or “synthetic” phenotypic states [4,5,8]. The synthetic phenotype is capable of synthesizing and secreting proteins associated with the extracellular connective tissue matrix, allowing them to proliferate and migrate to repair an injury under pathological conditions. The healthy adult vasculature, vessel tunica media specifically, consists predominantly of the contractile-state VSMCs. These cells are capable of reverting to a synthetic and proliferative phenotype in culture [4]. In addition to the expression of specific phenotype markers, synthetic VSMCs exhibit a characteristic “hill and valley” appearance, whereas contractile VSMCs appear more elongated and have spindle-like morphology [4]. VSMCs can de-differentiate, migrate, proliferate, and accumulate in the intima in response to vascular injury such as in atherosclerosis. 

This paper herein introduces an optimized protocol for isolating VSMCs from human umbilical cords. The protocol is a combination of both enzymatic and mechanical methods, which has not been previously reported. It also overcomes several limitations and yields a rich primary culture that can be obtained within 10 days and sub-cultured for 8–10 passages. These cells are characterized by their morphology, and the expression and presence of mRNA of some marker genes are verified by using reverse transcription polymerase chain reaction (RT-PCR).

## 2. Methods

### 2.1. Solutions

*Collection solution of the umbilical cord.* Phosphate-buffered saline (PBS) (Euroclone, Milan, Italy) supplemented with 3% penicillin/streptomycin (100× and 3% amphotericin B (100×) (Capricorn, Ebsdorfergrund, Germany).

*Complete medium for VSMCs.* Medium 199 (M199), 1% penicillin/streptomycin (Capricorn, Ebsdorfergrund, Germany), 1% L-glutamine (Euroclone, Milan, Italy), and 20% fetal bovine serum (FBS) (Citiva, San Diego, CA, USA), 0.1% insulin (Novo Nordisk, Bagsvaerd, Denmark), 0.2% glycine (Fischer Chemicals, Waltham, MA, USA), 0.03 mg/mL endothelial cell growth factor (ECGF) (Corning, NY 14831, USA), 0.1 mg/mL heparin (Sigma-Aldrich, St. Louis, MO, USA). Amphotericin (1%) was added to the medium in the first two passages of culture.

*Gelatin-coating solution.* Gelatin solution (0.5%) was prepared by adding 1 g of gelatin (Santa Cruz Biotechnology, Dallas, TX, USA) to 200 mL distilled water, then sterilized by autoclaving. Prior to cell seedling, 1 and 3 mL of the coating solution was used to coat 25 cm^2^ and 75 cm^2^ culture flasks, respectively. The flasks were kept still at 37 °C for 30 min, and then, the solution was aspirated before seedling.

*Collagen-coating solution.* Collagen solution (40 µg/mL) was prepared by adding 10 µL of 4 mg/mL type I collagen (Sigma-Aldrich, USA) to 1 mL distilled water. A total of 3 mL of the prepared collagen solution was added to 25 cm^2^ culture flasks and incubated for 1 h at room temperature or overnight at 4–8 °C. Excess fluid was removed and allowed to dry. The flasks were rinsed with PBS before adding the media.

### 2.2. Collection of Tissue Sample

Umbilical cord samples were obtained after Caesarean births to minimize contamination. The umbilical cord samples were collected in a sterile 50 mL conical tube containing the collection solution and maintained at 4 °C for 4–24 h. All procedures were carried out in a Class II laminar flow safety cabinet using an aseptic technique. Dissection equipment was thoroughly washed and kept sterilized by immersion in 70% ethanol or by autoclaving at 121 °C for 20 min. All procedures were approved by the Ethics Committee of Jordan University Hospital (decision no.: 182/2022, dated 28 June 2022).

### 2.3. Dissection of the Umbilical Cord

**Figure 1:**
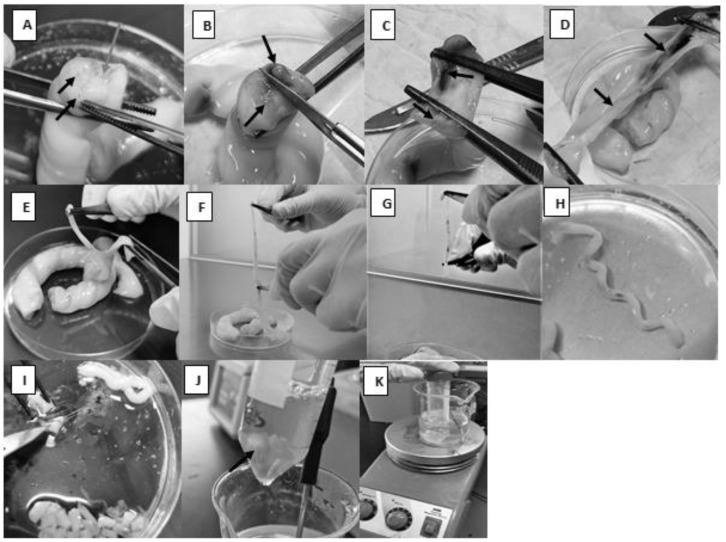
Dissection of the umbilical cord and preparation of the setup for artery enzymatic treatment. (**A**): The black arrows indicate the arteries and the white arrow indicates the vein. (**B**): A sagittal section that separates the two arteries from each other. (**C**,**D**): The two arteries with the surrounding tissues separated from each other. (**E**,**F**): Tissues around the artery removed. (**G**): Tunica adventitia is removed mechanically. (**H**): The spiral appearance of the artery. (**I**): Artery is being cut into 1–3 mm segments. (**J**): Arrow indicates the magnetic stir bar with the tissue pieces. (**K**): The whole setup for artery digestion.

Because the arteries are embedded helically in Wharton’s jelly, the umbilical cord was highly slimy and skiddy, and its dissection was relatively difficult. In the following section, we describe stepwise how to remove the tiny arteries and prepare them for VSMCs isolation:

1. The cord was removed from the collection solution, and a clean transverse cut was made across the two ends of the cord.
2. To obtain a higher yield of cells, a long segment of the cord (25–30 cm) was used.
3. The cord was cut into 3–5 cm long segments, and they were kept submerged in PBS in a sterile plastic Petri dish.
4. Using forceps, one segment was held in a way that allows for visualizing the two arteries and the vein (Figure 1A).
5. A sterile scalpel was used to make a sagittal section that separates the two arteries from each other so that the vein would be cut longitudinally (Figure 1B).
6. Each half was held with blunt, straight stainless-steel forceps, and they were ripped apart by pulling each half in the opposite direction (Figure 1C,D).
7. Using pointed forceps, the thick-walled artery was picked up and ripped carefully from the surrounding Wharton’s jelly (Figure 1E). It would not be a problem if the whole artery was not captured since it would be cut into smaller pieces at a later step. To make sure a segment of the artery, not Wharton’s jelly, would be specifically picked, note the spiral appearance of the artery (Figure 1H).
8. The isolated arteries were submerged in PBS in a new sterile Petri dish.

### 2.4. Cell Dissociation and Primary Culture of VSMCs

The modification described below overcomes the limitations stated in the introduction section. A sterile magnetic stir bar was used to create a mechanical force that enhances the detachment of a large number of cells in a short period. This was performed as follows:

1. Culture media and solutions were prepared and pre-warmed to 37 °C in a water bath.
2. As much surrounding connective tissue as possible was removed from around the arteries (Figure 1F); then, the arteries were immersed in PBS in a Petri dish to keep them moist.
3. To avoid contamination with other cell types, such as fibroblasts, adventitia was removed mechanically using forceps (Figure 1G). Note: the prevention of contamination with HUVECs is described in step 9 below.
4. The arteries were washed with PBS and transferred to a new container.
5. The arteries were cut into 1–3 mm pieces (Figure 1I).
6. The tissue pieces were placed in a sterile 50 mL conical tube.
7. A sterile magnetic stir bar was added to the conical tube and followed by approximately 10 mL of 0.3% collagenase type II to give a ratio of tissue to enzyme of 1:5 (*w*/*v*) (Figure 1J).
8. The conical tube was placed inside a 250 mL beaker containing 100 mL distilled water on a heating magnetic stirrer. The temperature was continuously set to 37 °C (Figure 1K). The mechanical movement of the magnetic stir bar inside the conical tube should enhance cell separation from the tissue.
9. After two hours, the solution was passed through a sterile sieve (an autoclaved stainless-steel strainer, 1 mm pore size) to remove any remaining tissue pieces. Based on our experience, when a 70 µm cell strainer (Corning, NY, USA) was used, the solution containing the cells would not pass through easily because it was highly viscous. In addition, the 2 h period was sufficient to yield a good number of VSMCs from tunica media without reaching tunica intima, which encompasses HUVECs that should be trapped by the strainer and, thus, prevents the contamination of the primary VSMC culture with HUVECs (see also the Supplementary Materials).
10. The cells were pelleted by centrifugation at 1120× *g* for 10 min at 4 °C.
11. Except for the last 2 mL, the supernatant was aspirated and discarded.
12. The pellet was resuspended in 5 mL of complete medium and plated in a gelatin-coated well (0.5 mL coating solution) of a 6-well plate.
13. The plate was incubated at 37 °C in a humidified incubator at 5% CO_2_ for 5 days. On the third day, and without disturbing the culture, 1 mL of complete medium was added gently to nourish the cells.
14. After the 5-day incubation period, the media with unattached cells were removed and refreshed with 2 mL fresh media. The red blood cells (RBCs) were discarded by gently washing the cultured cells with warm serum-free M199 medium, and the incubation was continued as usual until confluency.
15. The cells were split into a gelatin-coated culture flask as described below.

### 2.5. Subculturing

1. The culture medium was aspirated, and the cells were washed with pre-warmed, sterile PBS to remove any traces of serum.
2. Pre-warmed trypsin-EDTA solution was added (1 mL per 25 cm^2^ culture flasks or 2 mL for 75 cm^2^ culture flasks) to cover the cells, and the flask was placed in a humidified incubator at 5% CO_2_ and 37 °C for 2–4 min. The flask could be examined under a microscope to ensure complete cell detachment. This can also be facilitated by tapping the side of the flask. Note that VSMCs detach relatively slowly and, thus, may take a longer time to fully detach (approximately 10 min).
3. Serum-containing medium (double the amount of trypsin-EDTA solution) was added to stop the action of trypsin. To break up any cell clumps, the cell suspension was gently pipetted four to six times.
4. The cells were transferred into new gelatin-coated culture flasks at a split ratio of 1:3, and sufficient serum-containing medium was added to the new flasks (5 mL in a 25 cm^2^ flask and 10 mL in a 75 cm^2^ flask). Every 2–3 days, half of the culture medium was replaced by a fresh one until confluency. Note: the growth rate of the cells may decline and phenotypic changes become evident beyond passage 10, and therefore, further subculturing of the cells is not recommended.
5. For experimental protocols, growth medium can be replaced with either low-serum, low-ECGF, or serum-free medium for up to 24 h, but not later, since the cells may exhibit an altered apoptotic phenotype.

### 2.6. Characterization of VSMCs

The primary isolated VSMCs can be positively identified by their morphology and expression of VSMC markers. Morphologically, synthetic SMCs exhibit a characteristic “hill and valley” appearance, whereas contractile SMCs cells appear more elongated and spindle-like [4]. Other reports showed that human VSMCs had the capacity to form multicellular nodules at post-confluence [8]. Although nodule formation is not unique to VSMCs, this property distinguishes them from fibroblasts. As for cell markers, it has been reported that most VSMC markers are contractile proteins. Therefore, it is essential to maintain a contractile phenotype in vitro. Numerous modifications of the VSMC culture conditions have been described, including the use of a selective culture medium, serum reduction or deprivation, the addition of heparin, and collagen coating of culture ware surfaces [4]. In addition, no particular marker has been ascertained to be a sole indicator of a contractile VSMC phenotype. Therefore, the best practice is to assess a range of markers. Common VSMC markers include smooth muscle myosin heavy chain (SMMHC), α-smooth muscle actin, calponin, SM22 α, desmin, h-caldesmon, metavinculin, and smoothelin [4]. 

Reverse transcriptase quantitative polymerase chain reaction (RT-qPCR) was used to demonstrate the phenotypic characteristics of cultured VSMCs obtained from passage 3. Primers against α-smooth muscle actin, h-caldesmon, and calponin were used. The human housekeeping gene glyceraldehyde 3-phosphate dehydrogenase (GAPDH) was used to standardize the expression of the biomarkers (Table 1). The primers were selected according to Origine (Rockville, MD, USA) and purchased from Integrated DNA Technologies (Coralville, IA, USA). Total RNA was prepared using Riboex™ RNA isolation kit (GeneAll, Germany). The cDNA was prepared from mRNA using EasyScript^®^ All-in-one SuperMix (TransGen Biotech, Haidian District, Beijing, China). For RT-qPCR, PowerUp™ SYBR™ Green Master Mix (Applied Biosystems, Norwalk, CT, USA) was used. The delta–delta Ct method was used to calculate the relative fold gene expression of samples.

### 2.7. Cryopreservation

1. Cells at passage 3 or after were detached from a 75 cm^2^ culture flask (see step 2 of Section 2.5 above). Note: the cell content of a 75 cm^2^ flask can be frozen into 3 cryovials, if fully confluent.
2. Following centrifugation of the cell suspension for 10 min at 1120× *g*, the supernatant was aspirated, and the cell pellet was resuspended in 900 μL FBS for each cryovial with an additional 100 μL dimethyl sulfoxide (DMSO) and then transferred to a suitable cryovial.
3. The cryovial was then placed at 4 °C for 30 min, transferred to −20 °C for 30 min, and, finally, to −80 °C for 2 h before being immersed into liquid nitrogen for long-term (~6 months to 1 year) storage. Alternatively, a “Mr. Frosty” cryovial freezing chamber containing isopropanol was used and placed at −80 °C directly.
4. When re-culturing the frozen cells, they were thawed quickly, added to 5 mL M199 medium, and centrifuged at 1120× *g* for 10 min. The cell pellet was re-suspended and cultured as usual. Otherwise, the thawed cells were added to a 25 or 75 cm^2^ culture flask and left until they adhered to the surface (not more than 24 h) before renewing the medium.

## 3. Results

### 3.1. Cell Isolation and Cultures

**Figure 2:**
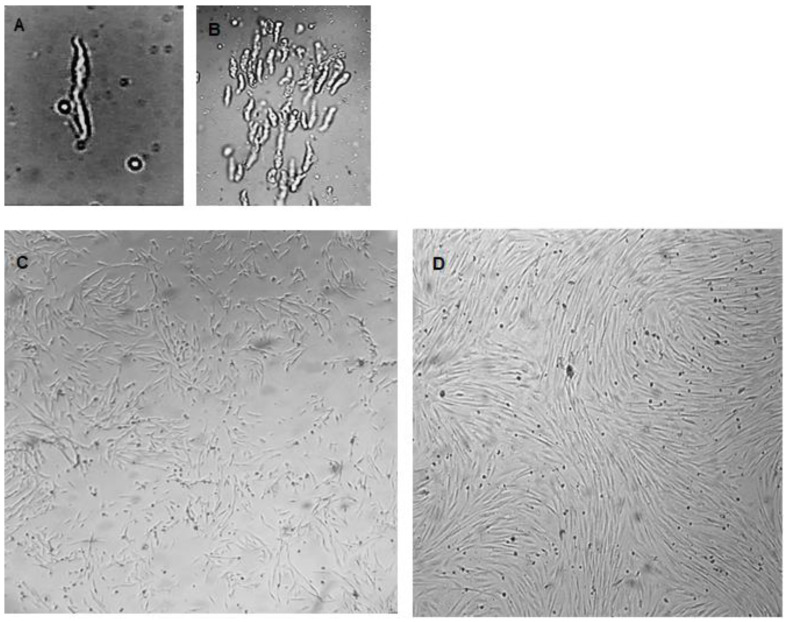
Primary cultured VSMCs were obtained using the described procedure. (**A**) A VSMC just after isolation and before attachment (magnification 400×), (**B**) VSMCs just after isolation and before attachment (magnification 100×), (**C**) synthetic VSMCs, (**D**) contractile VSMCs. (Magnification for (**C**,**D**) is 40×).

**Figure 3:**
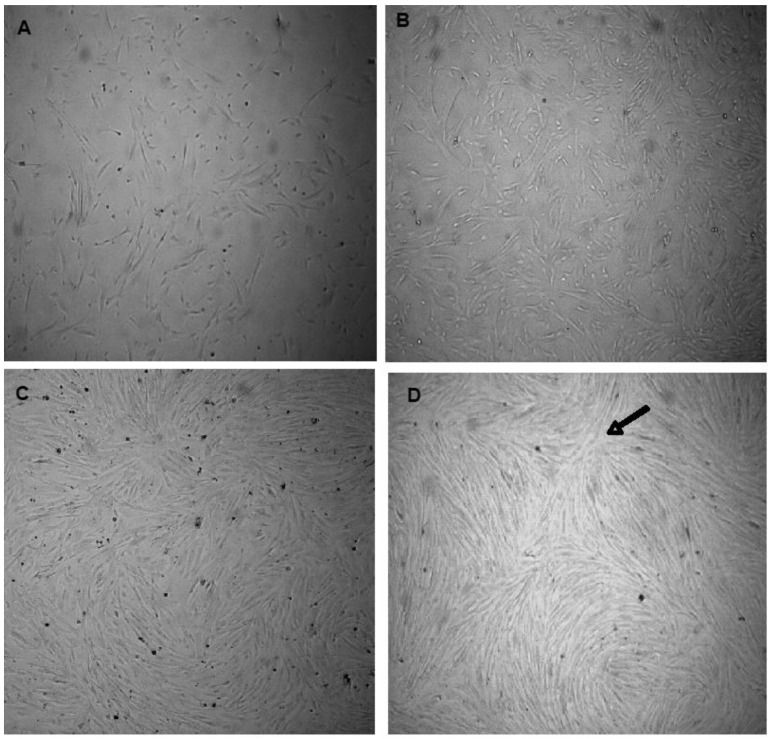
VSMCs at different cell densities. (**A**) 50% confluent VSMCs, (**B**) 70% confluent VSMCs, (**C**) 80% confluent VSMCs, (**D**) 90% confluent VSMCs with a nodule pointed at by the tip of the arrow. (Magnification is 40×).

The isolated cells were viable to develop primary cultures. Figure 2A,B show VSMCs before attachment at different magnifications. The synthetic cells and more elongated spindle-like contractile VSMCs are shown in Figure 2C,D, respectively. VSMCs at different confluences are presented in Figure 3A–D. The nodules could be observed (Figure 3D, at the tip of the arrow). 

### 3.2. Characterization

**Figure 4:**
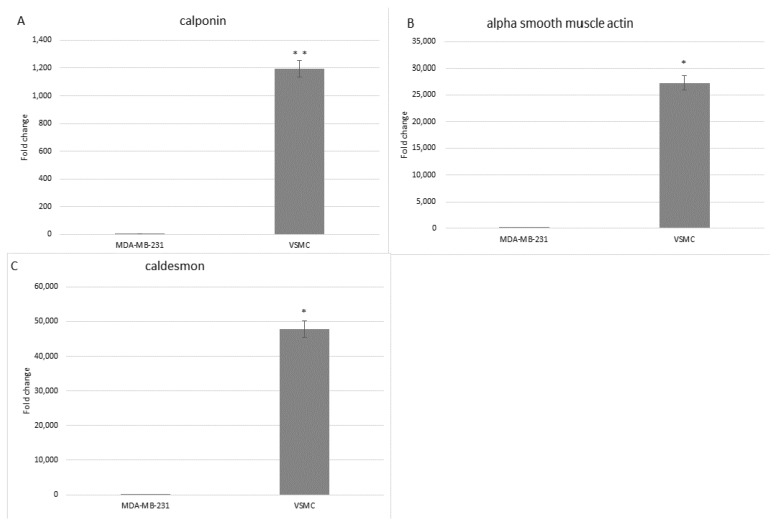
Fold change expression of mRNA for calponin (**A**), α-smooth muscle actin (**B**), and caldesmon (**C**) determined by RT-qPCR, in both isolated VSMCs and MDA-MB-231 breast cancer control cells. GAPDH was used as a reference gene. This experiment was performed twice (* *p* < 0.0001; ** *p* = 0.001).

In order to characterize the isolated cells at the molecular level, RT-qPCR analysis was performed using specific primers for three genes: α-smooth muscle actin, calponin, and caldesmon. Gene expression was normalized to that of GAPDH. Expression is presented as a fold difference relative to MDA-MB-231 breast cancer cells as the negative control. The contractile VSMCs cultured on collagen-coated flasks expressed the three markers at a much higher level than the control cells (Figure 4).

### 3.3. Isolation of HUVECs

The Supplementary file provides a brief description of an optimized protocol for the isolation of HUVECs. Supplementary Figure S1 illustrates the procedure. Cell morphology is shown in Supplementary Figure S2. The confirmation of the nature and purity of the cells is illustrated by staining the cells for von Willebrand factor and detection by using flow cytometry (Supplementary Figure S3).

## 4. Discussion

This study describes an optimized protocol for isolating VSMCs from a human umbilical cord by mechanical agitation, which has not been previously reported, combined with enzymatic digestion. Culturing immortal cancer cells is challenging, partly due to their need of specific concentrations of supplements and their limited lifetime in culture. Upon reviewing numerous protocols describing the isolation of VSMCs, we noted a lack of critical components in the media, which were enriched by a relatively high amount of FBS and supplemented with three growth factors (insulin, ECGF, and heparin) and, in addition to the antifungal agent, amphotericin [3,4]. Many published protocols used a mixture of proteolytic enzymes with a digestive cocktail of collagenases, elastase, soybean trypsin inhibitor, DNase, taurine, papain, trypsin, and other enzymes at variable combinations [5,9,10]. We efficiently and consistently isolated a large number of cells using only one enzyme, thus making our protocol simpler and cost-effective.

Another challenge in isolating VSMCs is contamination with other types of cells. In the presented protocol, contamination with fibroblasts was avoided by the mechanical removal of tunica adventitia, and contamination with endothelial cells was avoided by both proper timing of digestion and the use of a suitable strainer. Two hours of incubation with 0.3% collagenase does not digest whole blood vessels, leaving not only the tunica intima, which encompasses endothelial cells but also part of the tunica media, which exist as tissue pieces, intact and to be removed by sieving through an appropriately sized strainer. It is important to note that incomplete enzymatic digestion is compensated for by the use of a relatively long piece of umbilical cord to harvest a good yield of cells. 

The transformation of VSMCs from a contractile to a synthetic phenotype is a naturally occurring event during development and during vascular repair at sites of injury caused by, for example, hypertension, atherogenesis, and restenosis [4,8,11,12]. In atherosclerosis, VSMC phenotypic switching has a role in the initiation, stability, and, subsequently, the transfer of plaques [12,13]. Myointimal thickening and arterial occlusion arise from damage to the endothelium and exposure of VSMCs to circulating blood components such as oxidized lipoproteins [14]. In addition, conditions such as hyperglycemia and hyperlipidemia have been shown to transform the contractile phenotype of VSMCs into a synthetic phenotype, which plays a key role in the development of vascular diseases [4]. The stability of the lesion and its tendency to rupture was thought to be dependent on the amount and behavior of VSMCs within the lesion. Stable plaques contain abundant VSMCs and matrices, which form a thick fibrous cap above a lipid core. On the other hand, unstable plaques, which are more likely to rupture, contain few VSMCs, numerous inflammatory cells, and a thin cap [8]. Therefore, understanding the behavior of VSMCs in culture (i.e., contractile vs. synthetic) and the ability to control this behavior is important [13]. For example, for the characterization part, collagen was specifically used to coat culture ware instead of gelatin, and serum-reduced medium and heparin were added to ensure that the cells were in the contractile state. This was confirmed by the expression of α-smooth muscle actin, calponin, and caldesmon, all of which are specific for the contractile phenotype [4,11]. 

## 5. Conclusions

The protocol described herein for the isolation of VSMCs from human umbilical cords is easy and time- and cost-efficient. Isolated cells are useful model systems for understanding the mechanisms underlying many pathophysiological conditions.

## Figures and Tables

**Table 1:** Forward and reverse primers for vascular smooth muscle markers.

Gene	Forward Primer	Tm (°C)	Reverse Primer	Tm (°C)	References (Origene; CAT)
Calponin	CCAACGACCTGTTTGAGAACACC	58.3	ATTTCCGCTCCTGCTTCTCTGC	59.8	HP205213
α-smooth muscle actin	CTATGCCTCTGGACGCACAACT	59.3	CAGATCCAGACGCATGATGGCA	59.6	HP205437
Caldesmon	CTGTTCCTGCTGAAGGTGTACG	57.7	CCTACCTTCAAGCCAGCAGTTTC	58.0	HP234228
GAPDH	GTCTCCTCTGACTTCAACAGCG	57.4	ACCACCCTGTTGCTGTAGCCAA	61.3	HP205798

## Data Availability

All data generated and analyzed during this study are included in this article and its supplementary files.

## Supplementary Materials

The following supporting information can be downloaded at: https://www.mdpi.com/article/10.3390/mps6030054/s1, Figure S1: The setup used for isolating HUVECs, Figure S2: Primary cultured HUVECs, Figure S3: The isolated human umbilical vein endothelial cells (HUVECs) were sorted using flow cytometry.
