# Supplementary material for: A Modified Protocol for the Isolation, Culture, and Characterization of Human Smooth Muscle Cells from the Umbilical Cord
Source: Methods Protoc. 2023 May 26;6(3):54. doi: 10.3390/mps6030054 (PMC10304459; doi:10.3390/mps6030054)
Supplement: Supplementary file 1 [file mps-06-00054-s001.zip › mps-2356398-supplementary.pdf]

Supplementary procedure for isolating human umbilical vein endothelial cells (HUVECs)

Protocol

A simple, flexible, fast, widely used and reproducible method for an efficient isolation of viable HUVECs is the enzymatic method. HUVECs protocol will yield confluent HUVECs primary culture that can be obtained within a week and reliably sub-cultured for 5-7 passages. After obtaining the umbilical cord, make a clean transverse cut across one end of about 20 cm long segment of the cord. Insert a butterfly cannula into the vein (*Supplementary Figure 1 A*), secure the cannula in place using a sterile cord clamp or a disposable plastic surgical hemostat (*Supplementary Figure 1 B*), then fill a 20 ml syringe with warmed PBS and attach it to the tubing of the cannula at the other end, then use this solution to flush the vein with moderate pressure to get rid of blood clots, collect the waste in a beaker, if needed repeat washing again under sterile conditions. Lay cord on clean aluminum foil, fill the same syringe with 0.1% collagenase type II. Push collagenase into the vein until you see the first amount of solution exits the open end. Clamp the open end and fill with collagenase until there is moderate distention of vein (about 4 mL collagenase for a 20-cm piece of cord). Too much distention is not recommended. Submerge the cord in sterile PBS, cover with aluminum foil, incubate at 37°C for 15 min. Remove the cord from the incubator, and massage it gently to assist the detachment of cells from the vessel wall. Cut the end above the bottom clamp and collect the collagenase solution and cells in a 50 mL conical tube. Be sure to collect everything in the tube. Push the remaining collagenase through the vein, then fill the syringe with 20 mL warm PBS and inject through the vein with moderate pressure. Pellet the cells by centrifugation at 1120×g for 10 min. Aspirate supernatant (except for ~1-2 mL). Resuspend the pellet in 5mL complete medium and plate in a 25 cm^2 culture flask. (Complete medium is made of: Medium 199 (M199), 1% L-glutamine, 1% penicillin/streptomycin, and 10% fetal bovine serum (FBS), 0.03 mg/ml

endothelial cell growth factor (ECGF), 0.1 mg/ml heparin and 1% amphotericin, all are added to the medium in the first two passages of culture). Incubate at 37°C with 5% CO_2 overnight. Remove unattached cells and replace with fresh media. If there are many RBCs, wash once with warm serum free M199, continue incubating as usual until the plate is confluent (4-5 days). Split to a gelatin coated 75 cm^2 culture flask. Examine under a microscope. When properly differentiated, HUVECs acquire the so-called cobblestone phenotype. These cells are characterized by their morphology (*Supplementary Figure 2*), and the expression of the endothelial specific marker von Willebrand factor (vWF) that was analyzed by immunofluorescence experiment (*Supplementary Figure 3*).

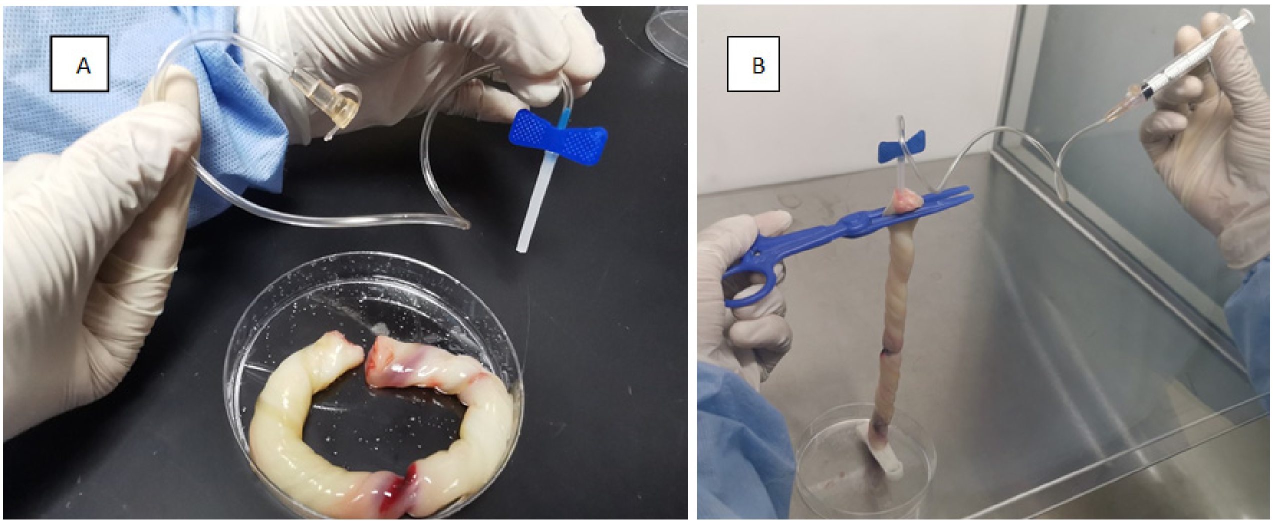

Supplementary Figure S1: The setup used for isolating HUVECs. A butterfly cannula is inserted into the vein without removing the white plastic part covering the needle (**A**) to prevent perforating the vein. After fixing the butterfly cannula, a 20-ml syringe filled with warm PBS is attached to the set up to flush the vein (**B**). The same syringe is filled with PBS, collagenase or any other solution injected through the vein.

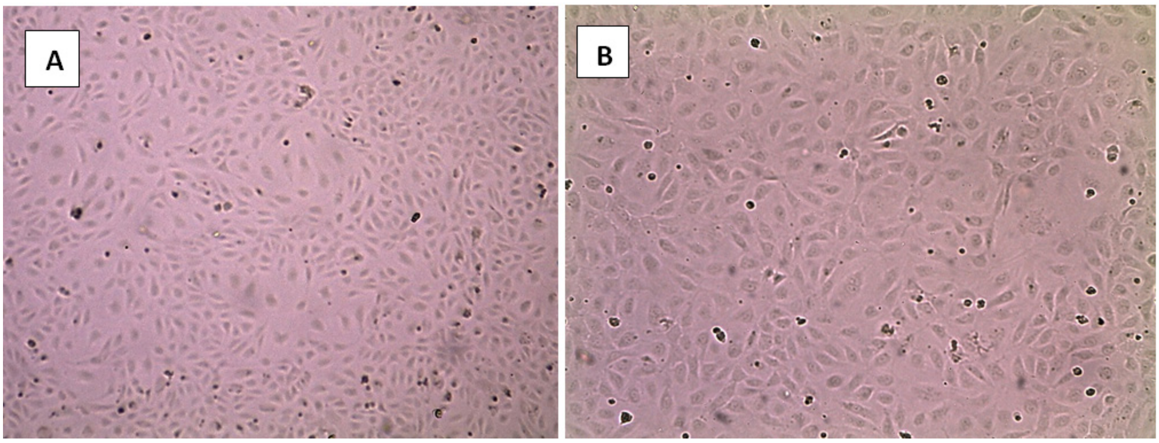

Supplementary Figure S2: Primary cultured HUVECs. **A** shows cells at a magnification of 40×, **B** shows cells at a magnification of 100×. In the two magnifications the "cobblestone" morphology is noted.

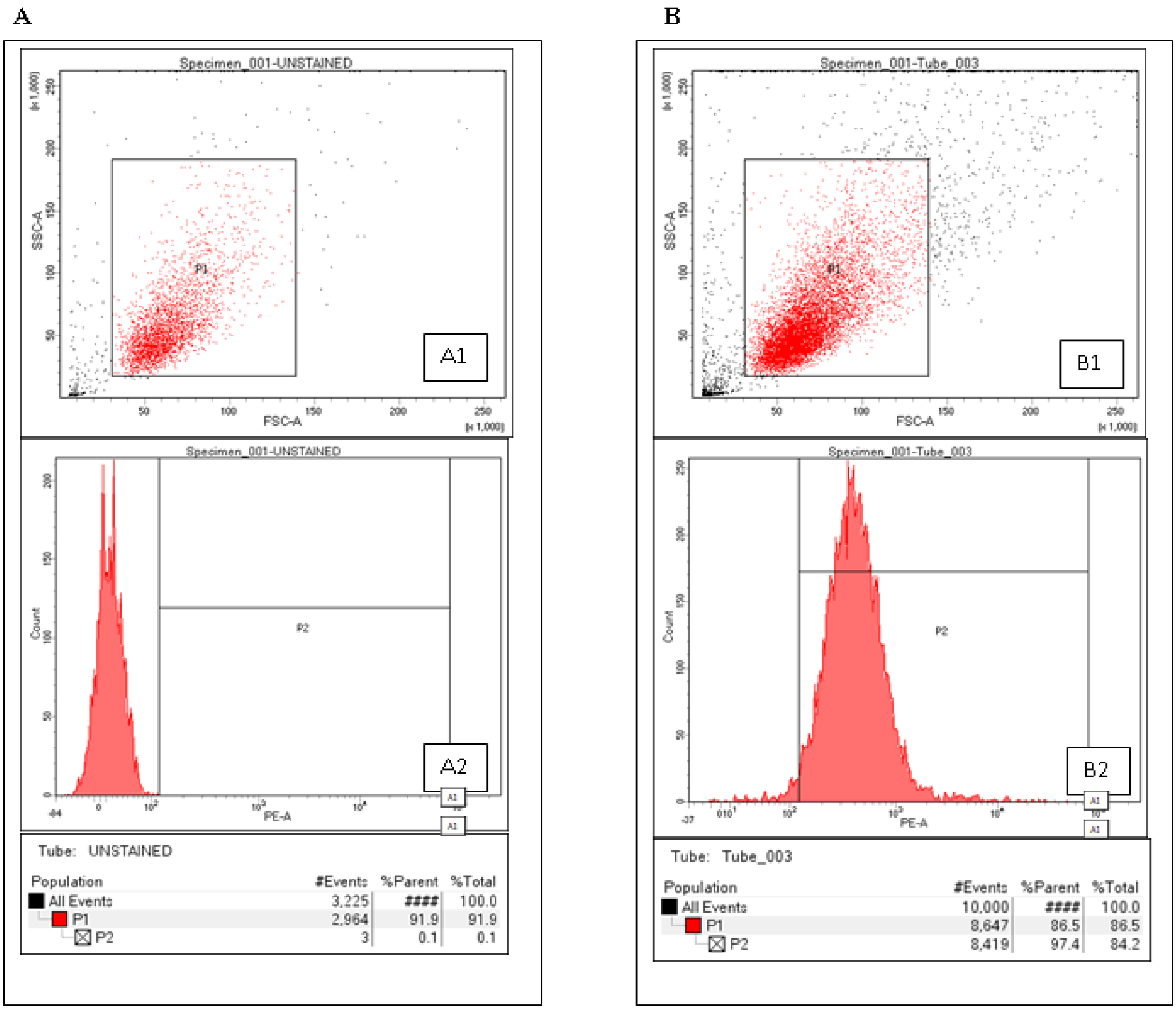

Supplementary Figure S3: The isolated human umbilical vein endothelial cells (HUVECs) were sorted using flow cytometry. Gate for sorting cells is illustrated in **A** (side and forward scatter **A1**, histogram **A2**). Cells that were stained using a labeled antibody against von Willebrand factor are shown in **B** (side and forward scatter **B1**, histogram **B2**). As shown in the Table in **B**, 97.4% of the cells were stained with the red dye indicating that the purity of HUVECs culture is more than 97%.
